# The politics of methodological reality practices of everyday life

**DOI:** 10.1177/13684310251404860

**Published:** 2025-12-08

**Authors:** Robert Braun, Richard Randell

**Affiliations:** 31339 Institute for Advanced Studies Vienna, Austria; 37748 Masaryk University, Czech Republic

**Keywords:** Ethnomethodology, ontography, apparatuses, automobility, infratextures

## Abstract

This article develops an analytic of ontopolitical apparatuses through an ethnomethodologically informed study of the methodological reality practices that constitute everyday existence. Building on John Law's call for methodological interference in “reality-making practices,” we foreground how being is stabilized not by foundational ontologies but by professional and mundane ontography—the performative inscriptions through which worlds are enacted and made accountable. Drawing on Dorothy Smith's sociology of extralocal relations of ruling and Karen Barad's agential realism, we argue that texts, practices, and materialities are not representational but discursive-material doings that reproduce the One-World World. Automobility serves as an exemplary technoscientific ontopolitical apparatus: a hegemonic formation that organizes subjects and objects into an ontocratic order, administering being through infrastructures and mundane reasoning—the infratextures of automobility. Its violence, routinely eventified as “accidents,” exemplifies the reflexive repair work through which disjunctures are normalized. We conclude by considering how alternative practices might interfere with ontographic forgetting and enable counter-ontopolitics.

In “On Sociology and STS,” John Law (2008, p. 639) asks if “we are well-served by the methodological reality practices that currently define the subject-matter, the representations, and the realities of sociology.” What is required, he suggests, are alternative methodological reality practices “to enact alternative […] versions of the real”; to “ask how our methods—appropriately extended—might productively interfere, both with one another, and with the reality-making practices that lie beyond the boundaries of sociology” (pp. 639–640). The reality-making practices that lie beyond the boundaries of sociology include not only the ontological practices of the natural sciences and the other social sciences, but also the everyday reality-making ontological practices by which everyday existence is routinely constituted and reproduced.

The focus of this article is the professional and mundane reality-making practices by which everyday existence is routinely constituted and reproduced; the reality practices by which the real is enacted and stabilized through the ontopolitical work that is performed by human and non-human agencies that “make [..] up the produced witnessability” of the world (Wiley & Garfinkel, 2019, p. 172). It explores alternative methodological reality practices that might effectively “interfere in the non-coherent structures of domination” (Law, 2008, p. 641).

In the following section we introduce an ethnomethodologically informed analytic (Garfinkel, 1967) to render visible the everyday reality-making practices through which the version of the real we happen to live in is constituted. That version of the real has been constituted as “out-there” by these methodological practices, the out-there being a world ostensibly populated by independently existing entities. We offer a deflationary ontographic analysis (Lynch, 2013) that examines locally enacted situated practices rather than a grand or global ontology (Woolgar & Lezaun, 2013). We examine how, within the world we inhabit, power is directed towards the administration of Being. This requires making explicit the tacit (performative) politics of *reality-making work*, asking how this version of the real came into existence, how it reflexively sustains and reproduces itself. Our argument intersects with Actor-Network Theory (ANT) and broader Science and Technology Studies (STS) work, and our reference to ethnomethodological sensitivity (Crawley et al., 2021) retains ethnomethodology's commitment to locally organized accountability while departing from ethnomethodology's avoidance of non-local relations in order to connect situated practices with wider ontopolitical apparatuses.

We then turn to a discussion of “automobility,” taking it as a case for understanding routine reality-making practices as they operate within ontopolitical apparatuses (Prozorov, 2017, p. 87). Due to its complexity and planetary reach, automobility serves as an exemplary case for understanding the processes by which Being is constituted and administered by and within ontological apparatuses (Braun & Randell, 2023).

We then consider alternative methodological reality practices and a new set of analytics to enact alternative versions of the real within both everyday existence and within scientific discourses. We round off with a discussion of our findings in which we reflect on the subversive potential of the new analytic and what this analytic means for instituting alternative reals (Esposito, 2021). We conclude with some reflections on how, by interfering with the reality-making practices that have constituted not just automobility but everything as existing “out-there,” such sensitivity might contribute to bringing about not a different One-World-World (OWW) (Law, 2015) but alternative worlds.

## Methodological reality practices of everyday life

Woolgar and Pawluch (1985, p. 215) proposed the term “ontological gerrymandering” to describe what they identify as the “selective application of relativism” within social constructivist accounts, by which some phenomena are held to be constructed and contingent whereas what are assumed to be stable background phenomena are rendered ontologically unproblematic, whose “truth value… is never questioned.” The ostensibly stable background phenomena are what are taken to be the real within a OWW ontology. An ever-present ontological gerrymandering permits the management of the practical tension between, on the one hand, a “dependable,” “reliable,” “non-fluid,” that is, “objective” explanans and, on the other hand, the social explanation(s) offered to make sense of the “social” construction (Woolgar & Pawluch, 1985, p. 224).

In line with the ontopolitical turn (Law & Urry, 2004; Mol, 1999; Mol et al., 2002) within STS, we recast the “weighty notion” signified by the term “ontology” as mundane everyday practice (Woolgar, 2022, p. 183). Gerrymandering is omnipresent and ubiquitous not only in the social sciences but also in everyday/everynight life. The gerrymandered—the world in its entirety and the individual objects contained within it—is the reality of mundane commonsense. Gerrymandering is not something that can be solved, rather it challenges us, Woolgar (2022, p. 183) argues, to think differently, to engage in “provocation”; to “give voice” (*pro vox*) “to things, people, situations and conditions”:

[T]o give voice to those entities which are neglected, overlooked or just reckoned to be mundane (that is, from latin *mundus,* those entities which have all the appearance of being of the world, that seem just the way they are). More especially perhaps, provocation means resisting the voices already associated with things, people, situations and conditions. Of always asking whether other alternative voices are possible.

Ontological gerrymandering is not merely a matter of epistemic selectivity or discursive bias, but a durable configuration of meanings, practices, infrastructures, and onto-epistemic techniques that together authorize particular ways of knowing and being while disqualifying or extinguishing others. Through ontological gerrymandering the material-discursive infrastructure of reality as it is currently lived is constituted and reproduced. Nothing less than commonsense, it is a hegemonic ordering in which it is assumed that the “out-thereness” of this ostensible singular reality is not a constituted out-there, that it is foundational, and that these claim can, and have been, apodictically established through metaphysical reasoning (see, for example, Bhaskar, 1978). The OWW ontology is the sedimented effect of this assumption, an order stabilized not only by conceptual frames but also by infrastructures of measurement, mobility, surveillance, and computation. Challenging this ontology requires attuning to a relational, emergent mode of becoming. It requires entertaining the possibility that it is never fully fixed, that the discursive-material practices that are embedded within broader extralocal regimes of ruling through which is routinely stabilized will always be temporary (Smith, 2001, p. 161).

An example is Dorothy Smith's (1999, 2001) ethnomethodologically informed feminist ontology, which treats “facts” and social order not as deterministic givens but as contingent accomplishments, simultaneously invention and interpretation, which are always situated within relations of power. This resonates with later feminist and queer theories while remaining ethnomethodologically attentive to the discursive/material. Barad (2003, p. 819) further radicalizes this rejection of representationalism in conceptualizing agency as emerging from intra-action rather than from interaction between already existing and already separated entities. Performativity for Barad is not the reduction of matter to discourse but a challenge to the privileging of language over practice, redirecting attention to the apparatuses through which realities and entities are enacted. Reality is not pre-given but emerges through practices, doings, and actions.

Ethnomethodology (EM) similarly directs attention to the local, embodied, and situational, to the ways in which coordinated doings in context generate meaning (Garfinkel, 1991, 2002; Maynard & Heritage, 2022). Context, situation, and place determine what can arise. Being is inherently indexical, contingent on the specific circumstances of its enactment, and reflexive, insofar as participants presuppose shared phenomenal awareness of the setting and, through their actions, reproduce localized versions of the OWW.

While ethnomethodology foregrounds the improvisatory creativity of actors—who continually adjust when rules prove inadequate—such creativity is always enmeshed in broader discursive-material regimes that limit what can be made sensible in the first place. As Smith (2005), departing from traditional ethnomethodology suggests, the enactment of order is never purely local. She urges attention to *text/reader conversations*: texts are not inert containers of meaning but materially replicable words or images that enter social relations only as they are taken up, interpreted, and enacted. Texts thus become actionable through practice, and it is the performative capacity of speech acts, which can never be disassociated from their denotative content, that organizes institutional relations of ruling (Smith, 1999, pp. 111–112). As Smith argues, post-structuralist discourse theory shows how meaning exceeds situated subjects, but in centering the self-referentiality of discourse it leaves unexamined the socially organized practices and relations—including those visible within discourse itself—that objectify subjects and bind them to the ruling relations (Smith, 1999, p. 80). Indexical and reflexive practices are therefore inseparable from sovereign operations. Local, accountable practices temporarily stabilize political order by drawing upon extralocal relations of ruling (Smith, 2001). Agency and order are not independent dimensions but co-constitutive, emerging in the dynamic interplay between situated coordination and the wider formations of power/knowledge that authorize, constrain, and make possible what can be enacted.

People's doings include the putting of texts in action. Texts do not operate by themselves; they require situated practices that animate them as coordinates of order. Smith shows how ordinary actors make institutional texts operative. An adequate empiricism may therefore exceed textual reading, combining attention to embodied interaction and extralocal relations of ruling, and to materiality as simultaneously and reflexively constituted and constitutive rather than backdrop. Such an empiricism is focused on *ontography*: it traces how worlds are inscribed into being.

Barad reminds us that such inscriptions are not merely representational but materially productive, enacting the very conditions of possibility. Professional ontography—such as, but by no means limited to, ontographic work performed by engineers, planners, lawyers, accountants, mathematicians, physicists and Chief Executive Officers—stabilizes relations through the creation of infrastructures, standards, and institutions (Braun & Randell, 2023). Mundane ontographies—everyday adjustments, recognitions, refusals—reproduce and reinforce (and sometimes unsettle) these inscriptions.

Ontography thus names both the expert labor of writing ontology into reality and the ordinary practices that sustain, reproduce or contest these writings. It is not description of what exists but enactment of what can hold. To follow ontographies is to track the indexical, reflexive, intra-active work through which the “ordinary world” is stabilized and politicized, routinely, again and again. To approach such an order with ethnomethodological sensitivity is to see how its taken-for-grantedness is locally and indexically maintained, while to address it ontopolitically is to situate this work within the broader sovereign operations through which reality is ruled. This double sensitivity—at once ethnomethodological and ontopolitical—enables us to grasp how methodological reality practices simultaneously enact the immediacy of everyday order and reproduce the instituted horizon of the OWW, within which other possible worlds, lives, and materialities are rendered marginal, invisible, unthinkable, or impossible.

Applied to the ontology we inhabit, an ethnomethodological sensitivity (Crawley et al., 2021) to ontological gerrymandering points to the challenge of giving voice to the beings that are ontologized away, that are negated and not afforded “becoming” (Barad, 2007). The STS provocation—“It Could Be Otherwise”—is a call to give voice through deconstructions of the governing onto-epistemology of the present. Informed by the “turn to ontology” in the social sciences (Escobar, 2007; Woolgar & Lezaun, 2013), recent work in the field of political ontology in Italian philosophy (Agamben, 2017; Esposito, 2011, 2021), and a resurgence of interest in the onto-epistemic implications of quantum theory (Barad, 2007; Braun, 2024; Voelkner & Zanotti, 2022), we make the case for a phenomenologically and ethnomethodologically informed approach as a way to recontextualize the methodological reality practices of the present.

### Professional and mundane ontography

Ontology, David Graeber (2015, p. 15) noted in the context of a discussion of the etymology and history of the word “ontology,” “is not a word for ‘being,’ ‘way of being,’ or ‘mode of existence,’ but refers instead to a discourse (*logos*) *about* the nature of being [*ontos*].” Much turns on what one takes “discourse (*logos*)” to be: as descriptive and denotative speech, or as inscriptive and performative speech *acts* (Austin, 1965; Derrida, 1988). A convenient term for the latter is the term “ontography,” if we take the Greek *gráfo*—to write—to mean writing in the Derridean sense of inscription, as *écriture* (Derrida, 2015; Rorty, 1982). As ethnomethodological studies have repeatedly demonstrated (Garfinkel, 1996), everyday practices are ontological (Pollner, 1987, pp. 135–147; Smith, 1999). They are practices by which the world is constituted, sustained and reproduced. “Ontology work” is the performative work of creating and sustaining worlds, entities, and their relations. It is work that is performed by those whom we might call “ontographers,” whose work is the work of performatively operationalizing “the world”: determining what it *is* and what *is not,* what kind of entities populate that *singular* world, which relations that world is made of. Through ontographic inscription practices, an ontology and the entities that are reflexively components of, and constitutive of, that ontology, are instituted. So conceptualized, ontology and ontography become one and the same, each a synonym of the other.

Along these lines Lynch (2019, p. 154) in an article “Ontography as the Study of Locally Organized Ontologies” proposed conceptualizing ontography as “intended to cover investigations that aim to describe the contingent and organizationally embedded work of social agents to propose, inscribe, or dispute particular ontological matters.” We rely on Lynch's deflationary, non-metaphysical account of ontography rather than any commitment to an underlying ontology, consistent with his insistence that ontography does not ground or justify large-scale metaphysical claims. The focus of his study is how historically and institutionally situated agents, such as judges and scientists involved in patent disputes, decide on the ontological status of entities such as adrenalin, plant fibers, genetically engineered organisms, and genetic sequences (Lynch, 2019). Engell and Siegert (2019) describe ontography as an “operative ontology,” simultaneously a concept, a procedure and a way of existence. In the arts it indicates esthetic techniques and technologies of drawing and registering (Engell & Siegert, 2019) and to general inscriptive strategies (Bogost, 2012). In Object Oriented Ontology (OOO) it refers to practices by which “things” are divided into real objects and sensual objects, as well as real qualities and sensual qualities (Harman, 2011). In anthropology, it refers to ontological auto-determination practices directed to the decolonization of thought, which Viveiros de Castro (2011, pp. 128–129) calls “speculative ontography.”

To inscribe the real through empirical discovery has, since the seventeenth century, become the task of science, both the natural and the social sciences (Shapin, 1984; Toulmin, 2013). Their *raison d’être* and purpose are to represent the properties and characteristics of “reality” through the employment of esoteric technoscientific practices and instruments (Ihde, 2009), such that the knowledge created by these practices will adequately represent “reality,” the world out-there, “as it ‘really’ is.” Through these practices which, within this ontology are held to be not inscriptive but denotative, “an ontology of one ontology,” a OWW, is enacted (Henare et al., 2007, pp. 9–10). It is an ontology that is routinely constituted and reproduced through, as Pollner (1987, p. 60) put it, “the commitment to the assumption that there is but one single world.”

It is an ontology wherein it is assumed as a matter of commonsense that “difference can only be a matter of different perspectives on, or different ways of perceiving, knowing, or representing that single reality” (Graeber, 2015, p. 18). The very terms, “perceiving,” “knowing,” “representing” are comprehensible only against the backdrop of a world experienced as out-there; as is the term “experienced” only comprehensible against the backdrop of a world that has been constituted as existing independently of the various ways it might be “experienced.” The very acts of “perceiving,” “knowing” and “representing” performatively inscribe the singular world that is experienced as existing out-there; simultaneously, the subject in-here is brought into existence (cf. Barad, 2003). It is an ontology that is built into the very fabric of the world (Wiley & Garfinkel, 2019, p. 172) and has transformed and now encompasses the entire planet. It has been constituted and is sustained through the routine practices of a diversity of agencies, one of which agencies is science (Garfinkel, 2022). Beyond the professional ontography described by Lynch, mundane ontography is the everyday inscriptive practices performed by ordinary members going about their routine daily activities. As Engell and Siegert (2019, p. 6) remark, “Ontologies do not wait for philosophers to be written and then descend on the world; rather, they are writing themselves as materially existing and effective operations.”

Analogous to what Pollner (1974, 1987) calls “mundane reason,” mundane ontography would be everyday performative inscriptions that enact the shared OWW ontology of the present, within which ontological disjunctures are othered, occluded, dissimulated and repressed (Law, 2004). It is the everyday inscription of entities and their determinate properties, the performative patterning of emergence and instantiation (Wallace, 2012) by which the OWW ostensibly independently existing ontology is reproduced. Dorothy Smith's concept of extralocal textual relations of ruling refers to the way institutional power is coordinated and extended across time and space through standardized texts—policies, forms, reports, regulations—that outlast local interactions and circulate beyond them and link the everyday practices of individuals, people's doings, to broader regimes of governance by organizing how actions must be read, recorded, and interpreted. We take ontography, both professional and mundane, as textual discursive-material doings: not the reduction of practice to language, nor the activation of texts by doing but the performative inscription of relations that stabilize certain realities while foreclosing others. Performativity, on this account, is not the privileging of words over matter but accountable, indexical, and reflexive discursive-material doings within contexts of power by which realities are continuously reinscribed.

Ontography “others away” alternative phenomena that cannot be readily entangled into observable relations (Barad, 2007) or made visible as “real patterns,” the non-random series of observables that make predictions about the world successful (Dennett, 1991). It is mundane reasoning in the ontological, by which intersubjective ontopolitical validation of the world (Massumi, 2015) is enacted through strategies for explaining away disjunctures, thereby permitting the continued maintaining of a shared OWW (Pollner, 1987).

Law (2008, p. 641) has argued that “methods that imagine the world to be relatively neat and tidy and try enact it in that way, are [..] seeking to stipulate and so to enact an order that is epistemologically mistaken, ontologically unrealistic, and politically obnoxious”; what Oksala (2010a, p. 457) has described as comforting naïve realism. Mundane ontography provides the certainty that the components of binaries such as imaginary and reality, culture and nature, human and animal, political and non-political, object and subject, are foundationally, essentially, always have been and always will be, *distinct;* that disjunctures are manageable—“for all practical purposes.”

### Ontopolitical apparatuses

Expanding on the Foucault's (1980, p. 194) definition of an apparatus and the range of apparatuses that Foucault investigated, Agamben (2009, p. 14) in “What is an Apparatus?” defined an apparatus (*dispositivo*) as: “[a]nything that has in some way the capacity to capture, orient, determine, intercept, model, control, or secure the gestures, behaviors, opinions. or discourses of living beings.” Comforting realism is made possible by the implicit neatness and tidiness of Cartesian/Newtonian apparatuses (Toulmin, 2013) that hold us captive (Wittgenstein, 1953 § 115) within both professional and mundane ontographic methods. A governing mechanism of ontopower (Massumi, 2015), apparatuses regulate who and what exists, with which properties, and within which hierarchy. Apparatuses are political in that they are the regulatory force of political being (Agamben, 2009; Foucault, 1980; Manderscheid, 2014; Oksala, 2010b). Rather than viewing apparatuses as totalizing regulatory forces (cf. Smith, 1999, p. 94), we approach apparatuses as intersubjectively organized ontopolitical text/reader relations—forms of coordination in which the intersection of being and power arises as an effect of the integration of the ruling relations, establishing subject positions within discourse from which experience is rendered knowable only externally and within an order of domination. This said, through the exercise of ontopower entities are inscribed into reality and come into being as the kind of entities that they are held to be. As Oksala (2010a, p. 464) has argued, “all ontology is always already political ontology.”

The capture Agamben refers to is in the form of subjectification and objectification, between the neatness and tidiness of the presupposed world “out-there” and the subject “in-here.” It is “the scission of being into essence and existence and the introduction of time into being” (Agamben, 2017, p. 1142). The scission is the ontological division between *zoē* and *bios*, between vegetative life (bare life) and qualified life (political life). It is a scission within humans, between “humans” and “animals,” between humans and “nature,” by which bare life and bare Being are constituted (Braun & Randell, 2023). These categories are all examples of ontological gerrymandering by which conditions of being are defined through the selective application of the mundane sceptical gaze (Woolgar, 2022, p. 185). It is an ontological scission that operates not solely at the level of ideas, as does, for example, Louis Althusser's (1971) concept of “ideological state apparatuses” but is inscribed into ontopolitical apparatuses which reflexively inscribe into the world (of which they are component elements) the scission (Agamben, 2017, p. 1142).

In his 1977 interview, Foucault (1980, p. 196) argued that, “The apparatus is always inscribed in a play of power.” We would add that ontopolitical apparatuses reflexively inscribe plays of power into reality. Ontopolitical inscription is enacted through “the said as much as the unsaid” (Foucault, 1980, p. 194), “the seen as much as the unseen” (Garfinkel, 2022, p. 58). In addition to Agamben's collection of apparatuses above, in *Homo Sacer* in the context of a discussion of Heidegger's inability to “resolve the problem of technology,” Agamben (2017, p. 601) refers to “the techno-ontological apparatus”; in the context of a discussion of “the epochal situation of politics and that of ontology” he refers to an “ontologico-political apparatus” (p. 1147); and in the context of a reading of Aristotle's *De anima,* he refers to a “metaphysical-political apparatus” (p. 1210). These three sets of adjectives point to a category of apparatuses—*technoscientific, ontopolitical apparatuses*—of which automobility is an exemplary case.

## Automobility as a case of ontographic methodological reality practices

*Qua* apparatus, automobility is more than a *Foucauldian* biopolitical apparatus (*dispositif*) of governmentality (Manderscheid, 2012, 2014). It is an apparatus through which sovereign ontopolitical power at planetary scale is exercised (Schmitt, 2003, 2005). Automobility has rhizomatically expanded across and transformed the entire planet, in the process destroying other ontologies, most obviously but not only, those of First Nations people (Anthony et al., 2024, p. 6). Automobility disrupted relational spatial ontologies in which land is a living, interconnected field of relations. Within the settler colony that is “Australia,” by segmenting Country with roads and privileging high-speed, individualized movement, automobility infrastructures replaced seasonal, collective, and custodial modes of mobility integral to First Nations ways of being (cf. Legacy et al., 2024). It is the OWW Cartesian/Newtonian ontology of occidental modernity in the material-semiotic form of an apparatus. It is ontopolitical in that the apparatus inscribes interconnected, systemic, constituted entities such as bodies, rulebooks, authorities, manufacturers, roads and steel; and everything else that is captured, temporarily or permanently, within the automobility apparatus: the sun, sunglasses, roadside flowers, insects and windscreens, animals and fenders, blue water navies and aircraft carriers, lithium production and slave labor, coal and electricity, death and injury, and more (Randell & Braun, 2022).

Across much of the automobility studies literature, automobility is understood to be deeply problematic (Anthony et al., 2024; Becker et al., 2012; Cass & Manderscheid, 2018; Hosseini & Stefaniec, 2023; Martens, 2016; Merriman, 2009; Miner et al., 2024; Newman, 2013; Seo, 2019; Sheller, 2018). A hegemonic apparatus, automobility is reproduced through its performative politics (Butler, 1990, 2015), its “acting in concert” whereby automobility creates its own public realm (Arendt, 1958; McGowan, 2022) while people's commonsensical doings reflexively re-enact the apparatus itself (Smith, 2005). That a world without automobility is virtually unthinkable (Braun & Randell, 2022a; Culver, 2018) attests to its material-ideological hegemony. Automobility does not just move people and things; it stabilizes reality itself. Through a recursive entanglement of discursive-material infrastructures (Hetherington, 2019) and embodied practices, automobility enacts a particular ontology—one in which roads, vehicles, drivers, pedestrians, liability, and “accidents” appear as naturalized, separable, and determinate entities. This ontology is not prior to the apparatus; it is continuously performed into being. This performance unfolds through the interplay between professional ontography and mundane ontography by which extralocal relations of ruling are continuously enacted in everyday life (Smith, 1999, 2005).

While mobilities scholarship has been invaluable in foregrounding automobility as a central theme of critical inquiry (Merriman, 2009; Sheller & Urry, 2006, 2016; Urry, 2004), much of this work tends to reify automobility as a stable object—a sociotechnical “system” or “assemblage” whose elements, boundaries, and effects are presumed to pre-exist description and analysis. In a move akin to Law and Mol's (2002) “utopian” analysis of railway accidents—as locally enacted, discursive-material *and* silently tinkered, performed rather than causally dissectible events—we approach what are called “accidents” within automobility as ontographic productions rather than as stable objects presupposed by much of mobilities theory. Our approach is concerned less with describing automobility as an already-constituted thing and more with understanding the performative practices through which automobility becomes real. Mobilities scholarship on accidents offers a methodological orientation to the situated production of events, which we use to explore how automobility's harms—both mundane and catastrophic—are ontographically accomplished. Drawing on Barad's (2007) agential realism and Dorothy Smith's (2005) institutional ethnography, we position automobility not as a discrete socio-technical domain but as a discursive-material infrastructural locus where worlds are enacted. It is precisely in the intra-actions between professional and mundane ontographies—the (discursive-material) infrastructurally mediated, situated, embodied, and interpretive performances of everyday/everynight life—that automobility stabilizes its ontological order. Automobility, for us, is the object of analysis inasmuch as it is the site of ontographic work, where professional and mundane performances recursively reproduce, transform, reinforce, and reproduce the extralocal relations of ruling that make automobility both intelligible and normalized.

For Smith, institutional power is coordinated and extended across space and time through replicable texts that circulate beyond the sites of their production. In respect to the subject at hand, these texts include policies, forms, traffic codes, insurance claims, technical standards and so forth. The professional ontographers of automobility—engineers, planners, insurers, safety statisticians, lobbyists, automakers and more—perform the work of inscribing texts, stabilizing relations through the creation of standardized, transportable forms. Yet these extralocal relations of ruling are never operative on their own. They depend on the situated practices of ordinary members, whose everyday enactments animate, adapt, and, most importantly, reproduce them. Mundane automobility ontography is the performative accomplishment of automobility in the embodied routines of walking, driving, cycling, navigating, repairing, and narrating, as well as of socialities such as work, home and entertainment. Paraphrasing Kallinikos’ (1995 quoted in Smith, 2001) theorization of textually mediated, detached forms of social relations as “archi-texture,” we rename automobility infrastructures as automobility* infra-textures*: discursive-material, mediated forms of ruling relations recursively reinscribed by mundane automobility performances.

This recursive relationship is central to automobility's ontopolitical power. Professional ontography provides a grammar of governance beyond actual texts: professional ontographers inscribe reality into discursive-material infrastructure; mundane ontographers render that grammar operational in local contexts while also subtly transforming and re-inscribing it. Automobility is one of the ontopolitical apparatuses that regulates the naming of any and all entities one might encounter; the categorizing of the properties of entities; controlling the placement and hierarchical position of entities, such as human/animal, human/nature, driver/pedestrian (Adorno, 2010, p. 40), reality/representation; determining what reality “is” and what “it” is not. In short, determining and constituting the ontology not only of automobility but of contemporary existence itself. Not least because it is through the exercise of ontopolitical power that it is possible to constitute automobility as a singular and spatially circumscribed phenomena, yet automobility is a spatial order that is everywhere (Braun & Randell, 2022b; Randell et al., Forthcoming). Prescribing congealments of relations by professional ontographers is a mechanism of power that regulates the properties of entities, as already always experienced and as determinate and individualized.

Power is exercised through the creation and regulation of space (road, parking lot, lanes), of time (rhythms, speed), through endowing only the human with agency (responsibility, guilt). It is enforced with the aid of complex systems for imputing causality (statistics, insurance, fines). Through this discursive and material-semiotic congealing of relations, the ontological normalization that is “out-thereing” is achieved. As Smith argues, the authority of extralocal ruling relations is secured by their ongoing activation in lived practice. Smith’s (2001) concern is with text-based institutional setups becoming alive and locally real by indexical accomplishments. Similarly, professional and mundane automobility ontographies reflexively weave the infrastructure into reality as mundane, insignificant, commonsensical and as ontologically singular and out-there (Pollner, 1987).

Automobility reality is a continuous ontopolitical performative interaction between discursive-material entities that are assumed to be “found” *in* the world and that *inter-act* with one another; rather than entities and a world which through intra-action come into being (Barad, 2003). The continued reproduction of automobility is dependent upon the work of mundane ontography—agents who take for granted what has been constituted by professional ontographers: that automobility is “real” in accord with how “real” is understood within a traditional OWW ontology. It is a constituted real defined in terms of its most visible component—“the car.” That reproduction is achieved through the ontology work of a myriad of professional and mundane automobility ontographers, working relentlessly to inscribe textually mobilized extralocal relations of ruling such as routinized “causal attributions,” “responsibility,” “effects,” “sequences,” “culpability,” “sustainability,” and so forth. The real is constituted as a sequence of spatiotemporally situated, contingent, materially and textually preformulated and congealed relations of ruling, which mundane ontography continuously re-enacts.

Within automobility, our very “competence as members of society” (cf. West & Zimmerman, 1987, p. 126), indeed our very lives, is hostage to the continued reproduction of automobility. One of the ways by which that competence is attested to is through the skilful correcting of disjunctures, achieved in intersubjective situated praxes of cognitive, practical and interactional work in meaning production (Pollner, 1974, pp. 53–54). “The accident” is such a disjuncture. Virilio (2007, p. 10) observed that “to invent the train is to invent the rail accident of derailment […] to invent the family automobile is to produce the pile-up on the highway.” What Virilio called “the integral accident” is everywhere in the sense that the accident is not contingent but inseparable from the apparatus. Automobility violence is not only integral but constitutive of automobility. Everywhere where automobility first appears, it is through violence that streets are cleared of pedestrians, children and other entities that hinder automobility movement (Lamont, 2012; Norton, 2008), and it is violence that keeps streets open as thoroughfares for automobiles (Braun & Randell, 2022b).

Automobility violence, routinely rendered witnessable as an “accident,” exemplifies what Pollner terms a reality disjuncture: a rupture in the seamless flow of everyday life. Such incidents threaten the ontological stability of automobility, requiring immediate repair. This repair is achieved through mundane ontography—the performative re-inscription of reality into intelligible and accountable form. In Dorothy Smith's terms, this involves textual coordination: reports, statistics, and institutional classifications that translate local experience into administratively usable knowledge. Central to this ontography is *eventification*, the conversion of the integral violence of automobility into discrete “accident events.” Although death and injury are constitutive and endemic, each incident is framed as contingent, bounded, and exceptional. Through witness testimony, police categorization, and insurance documentation, violence is transformed into an event localized in time, space, and causality (Bucsuházy et al., 2020; Zhang et al., 2018). Explanations citing driver error, road conditions, or vehicle malfunction accomplish the interactional work of containment, rendering the accident both intelligible and governable (Chenisbest et al., 1998; Elvik, 2004; Law & Mol, 2002).

This process restores the coherence of the (social) world by recasting constitutive harms as anomalous disruptions. Eventification, the mode of entangling local performances and extralocal relations of ruling into a spatio-temporal fix, occludes the systemic spatial and temporal ubiquity of automobility violence, reallocating agency to individual drivers thereby occluding the violence that is an integral property of the automobility apparatus itself. In ethnomethodological terms, members achieve the normalization of rupture; in Smith's sense, institutional texts align these member-methods with broader ruling relations that sustain automobility. The accident therefore appears not as integral but as contingent. Through ontographic practices of eventification, disjuncture is folded back into commonsense everyday order, preserving the hegemony of automobility.

Tracing these methodological reality practices allows us to move beyond descriptive accounts of automobility's sociotechnical dominance toward an analysis of ontopolitical power. Automobility is neither a neutral technology nor a neutral infrastructure for movement but a planetary-scale configuration of performative ontographies. It organizes the definition of entities, the distribution of agency, the stabilization of normative relations, and the embedding of epistemic authority into everyday sense-making. The interplay between professional and mundane ontography makes its order durable yet dynamic, ensuring its reproduction while allowing for iterative transformation. It is through this recursive coupling—between the ruling discursive-material infrastructure and lived performances, between institutional designs and embodied improvisations—that automobility achieves its ontographic hegemony, stabilizing a singular ontology of mobility while rendering alternatives unimaginable.

## Ontographic forgetting

Worlds, their component entities, and the relations between their component entities, are constituted, sustained and reproduced through ontology work. Ontological gerrymandering and performative ontology work involves transformative forgetting, ignoring the politics of ontological transformations, that other worlds, other entanglements are not only possible but might also be “real” (Braun, 2024). As Esposito (2020, p. viii) has argued, “every philosophical definition of Being has presuppositions, and effects, of a political nature.” One of those political effects is the ontopolitical forgetting of other worlds, other potentialities, non-human entanglements, less-than-entities that as yet have no “voice” (Raymond et al., 2025). It is one mode of enactment by which the real is constituted as universal and foundational, as hegemonic commonsense, both within mundane existence and the philosophy of science. It is the creating and reproducing of the ontology of the present through routine ontology work by human and non-human agents. As Oksala (2010b, p. 464) has argued, “the ontological order of things” is constituted through political struggle. Her description of ontology as “politics that has forgotten itself” suggests, however, that this forgetting in the realm of the ontological passively occurs. Rather, it is through the professional and mundane work of *ontographic forgetting* that the ontology that is the OWW is constituted as devoid of politics, as apolitical.

Pollner (1987, p. 58) observed that in our “attempts to discern the structure of mundane reasoning… it is difficult to obtain an ironic view because the mundane idiom… so deeply permeates scientific and common-sense thought.” “Representation, metaphysical individualism, and humanism,” Barad (2007, p. 134) similarly observes, have an extraordinarily “powerful grip on contemporary patterns of thought.” A foundational assumption of scientific and mundane commonsense reasoning is the existence of an objective world out-there that is separate from the epistemic subject. The task, Pollner (1974, p. 37) remarks, is to imagine “how one could reason otherwise.” As Husserl (1970, p. 137) once intimated, the challenge is not a purely intellectual challenge of reasoning differently, but is an ontological challenge requiring a “complete personal transformation,” comparable to, as he put it, “a religious conversion,” which, he added, “bears within itself the significance of the greatest existential transformation which is assigned as a task to mankind as such.” Or, as Leary (1970, p. 13) once put it, it would be akin to waking up from a “long ontological sleep.”

We have suggested that ontology work is the performative work of creating and sustaining worlds, entities, and their relations. So conceptualized, there is no such thing as an ontology that is out-there, waiting to be described in its independent plenitude. Ontographic ethnomethodological sensitivity points to reals always being performatively constituted, although not necessarily uniquely and solely by humans. Apparatuses are heterogeneous assemblages, instantiating patterns, structures, consisting of human and non-human emergent entities, inscribed by their reflexive members, in specific entanglements that fix entities and their relations as a particular “way of seeing” that is called “reality” (Lohrey & Boreham, 2020). Apparatuses are recast as “ontopowerful” (Massumi, 2015); they create the conditions of possibility of being, whether of discourse or silently invested material practice, forms of spatiality, temporality, matter and relations that define (“local,” “observable,” “real”) conditions of political being. Such apparatuses create congealments in and by which relations of exteriority, connectivity, and exclusion are configured when “observed” locally by the observer (Barad, 2007). One task is to give voice through iterative reconfigurings to less-than-entities, more-than-humans and the non-human. In other words: to render ontologically significant that which is insignificant within the performances that are the ontology of the present.

## Conclusion

Our aim in this article has been to open up a space for thinking the otherwise, identifying ruptures, developing directions towards a *counter ontopolitics* to the ontological gerrymandering through and by which the OWW is constructed. The point of our investigation was to expand on John Law's question quoted in the introduction. To bring to the table alternative research perspectives to develop potential forms of ontopolitically destituent resistance that would challenge the comforting faith in the neat commonsense realism and the commonsense conviction that the world could not be otherwise, that There Is No Alternative—TINA—which absence of alternatives is one of the central hegemonic properties of the ontology. As Woolgar and Lezaun (2013, p. 323) argue, “The purpose of researching ontology… [is] not… to arrive at a better formulation of the reality of the world, or of the ways in which the world is real, but to interfere with the assumption of a singular, ordered world, and to do so by re-specifying hefty meta-physical questions in mundane settings and in relation to apparently stabilized objects.” An ontologically and ethnomethodologically focused reformulation of Marx's Eleventh Thesis on Feuerbach, it represents a shift not just in the manner of interpretation but in how we might constitute an ontological otherwise.

It is our hope that this article might contribute towards better understanding the practices by which the world has been made fit both for sovereign state and technoscientific power (Law, 2008, p. 640). With this we move towards the outer rims: “the processes constituting the discursive and institutional spaces within which particular practices are deployed” (Pollner, 1991, p. 376). The central challenge we face is that of how to reflexively engage with ruling relations and destabilize the comforting real, the indubitable reality of which, when seen from the vantage point of the technoscientific subject and experienced within ontopolitical apparatuses that are continuously inscribed by professional and re-enacted by mundane ontography, is nothing less than commonsense. Such a critique is a prerequisite for constituting not *an* alternative world—one more One-World-World—but alternative *worlds.* Worlds which, to paraphrase Arendt (1994, p. 233), would be fit for human, non-human and more-than-human habitation, wherein voice might be given to the more-than-human and to less-than-entities.

## References

[bibr1-13684310251404860] Adorno T. W. (2010). Minima Moralia: Reflections on a damaged life. Verso.

[bibr2-13684310251404860] Agamben G. (2009). What is an apparatus and other essays. Stanford University Press.

[bibr3-13684310251404860] Agamben G. (2017). The omnibus homo sacer. Stanford University Press.

[bibr4-13684310251404860] Althusser L. (1971). Ideology and ideological state apparatuses (notes towards an investigation). In Lenin and philosophy and other essays (pp. 121–173). Monthly Review Press.

[bibr5-13684310251404860] Anthony T. Sherwood J. Blagg H. Tranter K. (2024). Unsettling colonial automobilities: criminalisation and contested sovereignties (1st ed.). Emerald Publishing.

[bibr6-13684310251404860] Arendt H. (1958). The human condition. Chicago University Press.

[bibr7-13684310251404860] Arendt H. (1994). *Eichmann in Jerusalem: A report on the banality of evil*. Penguin Classics. (1965).

[bibr8-13684310251404860] Austin J. L. (1965). How to do things with words. Oxford University Press.

[bibr9-13684310251404860] Barad K. (2003). Posthumanist performativity: Toward an understanding of how matter comes to matter. Signs: Journal of Women in Culture and Society, 28(3), 801–831. 10.1086/345321

[bibr10-13684310251404860] Barad K. (2007). Meeting the universe halfway: Quantum physics and the entanglement of matter and meaning. Duke University Press.

[bibr11-13684310251404860] Becker U. J. Becker T. Gerlach J. (2012). *The true costs of automobility: External costs of cars overview on existing estimates in EU-27* .

[bibr12-13684310251404860] Bhaskar R. (1978). A realist theory of science. Humanities Press.

[bibr13-13684310251404860] Bogost I. (2012). Alien phenomenology, or what it’s like to be a thing. University of Minnesota Press.

[bibr14-13684310251404860] Braun R. (2024). Radical reflexivity, experimental ontology and RRI. Journal of Responsible Innovation 11(1). 10.1080/23299460.2024.2331651

[bibr15-13684310251404860] Braun R. Randell R. (2022a). Post-Automobility futures: Technology, power, and imaginaries. Rowman & Littlefield. https://rowman.com/ISBN/9781538158852/Post-Automobility-Futures-Technology-Power-and-Imaginaries

[bibr16-13684310251404860] Braun R. Randell R. (2022b). The vermin of the street: The politics of violence and the nomos of automobility. Mobilities, 17(1), 53–68. 10.1080/17450101.2021.1981118

[bibr17-13684310251404860] Braun R. Randell R. (2023). The political ontology of automobility. Mobility Humanities, 2(1), 7–22. 10.23090/MH.2022.07.1.2.007

[bibr18-13684310251404860] Bucsuházy K. Matuchová E. Zůvala R. Moravcová P. Kostíková M. Mikulec R. (2020). Human factors contributing to the road traffic accident occurrence. Transportation Research Procedia, 45, 555–561. 10.1016/j.trpro.2020.03.057

[bibr19-13684310251404860] Butler J. (1990). Gender trouble: Feminism and the subversion of identity. Routledge. http://www.dawsonera.com/depp/reader/protected/external/AbstractView/S9780203902752

[bibr20-13684310251404860] Butler J. (2015). Bodies that matter: On the discursive limits of sex. Routledge.

[bibr21-13684310251404860] Cass N. Manderscheid K. (2018). The autonomobility system: Mobility justice and freedom under sustainability. In Cook N. Butz D. (Eds.), Mobilities, mobility justice and social justice (pp. 101–115). Routledge.

[bibr22-13684310251404860] Chenisbest B. Jähn N. Le Coz J.-Y. (1998). *European Accident Causation Methodology (EACS)* .

[bibr23-13684310251404860] Crawley S. L. Whitlock M. Earles J. (2021). Smithing queer empiricism: Engaging ethnomethodology for a queer social science. Sociological Theory, 39(3), 127–152. 10.1177/07352751211026357

[bibr24-13684310251404860] Culver G. (2018). Death and the car: On (auto)mobility, violence, and injustice. ACME: An International Journal for Critical Geographies, 17(1), 144–170. https://doi.org/10.14288/acme.v17i1.1580

[bibr25-13684310251404860] Dennett D. C. (1991). Real patterns. The Journal of Philosophy, 88(1), 27–51. 10.2307/2027085

[bibr26-13684310251404860] Derrida J. (1988). Limited Inc. Northwestern University Press.

[bibr27-13684310251404860] Derrida J. (2015). *Of grammatology* .

[bibr28-13684310251404860] Elvik R. (2004). *Laws of accident causation* 18th ICTCT workshop. https://www.ictct.net/wp-content/uploads/18-Helsinki-2005/ictct_document_nr_57_S7_Elvik.pdf

[bibr29-13684310251404860] Engell L. Siegert B. (2019). Editorial. ZMK Zeitschrift für Medien- und Kulturforschung: Ontography, Nr. 1, 5–11. 10.25969/mediarep/18716

[bibr30-13684310251404860] Escobar A. (2007). The ‘ontological turn’ in social theory. A Commentary on ‘Human geography without scale’, by Sallie Marston, John Paul Jones II and Keith Woodward. Transactions of the Institute of British Geographers, 32(1), 106–111. 10.1111/j.1475-5661.2007.00243.x

[bibr31-13684310251404860] Esposito R. (2011). Immunitas: The protection and negation of life (English ed.). Polity. Publisher description. http://www.loc.gov/catdir/enhancements/fy1210/2012382541-d.html Table of contents only http://www.loc.gov/catdir/enhancements/fy1210/2012382541-t.html

[bibr32-13684310251404860] Esposito R. (2020). Pensiero istituente : tre paradigmi di ontologia politica. Einaudi.

[bibr33-13684310251404860] Esposito R. (2021). Instituting thought: Three paradigms of political ontology. Polity Press.

[bibr34-13684310251404860] Foucault M. (1980). Power/Knowledge: Selected interviews and other writings (C. Gordon, Ed.). Pantheon Books.

[bibr35-13684310251404860] Foucault M. (1980). Power/knowledge: Selected interviews and other writings, 1972-1977. Pantheon Books.

[bibr36-13684310251404860] Garfinkel H. (1967). Studies in ethnomethodology. Prentice-Hall.

[bibr37-13684310251404860] Garfinkel H. (1991). Studies in ethnomethodology. Polity Press.

[bibr38-13684310251404860] Garfinkel H. (1996). Ethnomethodology's program. Social Psychology Quarterly, 59(1), 5–21. 10.2307/2787116

[bibr39-13684310251404860] Garfinkel H. (2002). Ethnomethodology’s program: Working out Durkheim’s aphorism (A. W. Rawls, Ed.). Rowman & Littlefield.

[bibr40-13684310251404860] Garfinkel H. (2022). Harold Garfinkel: Studies of work in the sciences. Routledge. 10.4324/9781003172611

[bibr41-13684310251404860] Graeber D. (2015). Radical alterity is just another way of saying “reality”: A reply to Eduardo Viveiros de Castro. HAU: Journal of Ethnographic Theory, 5(2), 1–41. 10.14318/hau5.2.003

[bibr42-13684310251404860] Harman G. (2011). The quadruple object. Zero Books.

[bibr43-13684310251404860] Henare A. J. M. Holbraad M. Wastell S. (2007). Thinking through things: Theorising artefacts ethnographically. Routledge/Taylor & Francis Group. Table of contents only http://www.loc.gov/catdir/toc/ecip0617/2006022250.html Contributor biographical information http://www.loc.gov/catdir/enhancements/fy0653/2006022250-b.html Publisher description http://www.loc.gov/catdir/enhancements/fy0653/2006022250-d.html

[bibr44-13684310251404860] Hetherington K. (2019). Infrastructure, environment, and life in the anthropocene. Duke University Press.

[bibr45-13684310251404860] Hosseini K. Stefaniec A. (2023). A wolf in sheep's clothing: Exposing the structural violence of private electric automobility. Energy Research & Social Science, 99, 103052. 10.1016/j.erss.2023.103052

[bibr46-13684310251404860] Husserl E. (1970). The crisis of European sciences and transcendental phenomenology. Northwestern University Press.

[bibr47-13684310251404860] Ihde D. (2009). Postphenomenology and technoscience: The Peking University lectures. State University of New York Press.

[bibr48-13684310251404860] Kallinikos J. (1995). The architecture of the invisible: Technology is representation. Organization: The Interdisciplinary Journal of Organization, Theory, and Society, 2(1), 117–140. https://doi.org/10.1177/13505084952

[bibr49-13684310251404860] Lamont M. (2012). Accidents have no cure! Road death as industrial catastrophe in eastern Africa. African Studies, 71(2), 174–194. 10.1080/00020184.2012.702964

[bibr50-13684310251404860] Law J. (2004). After method: Mess in social science research. Routledge.

[bibr51-13684310251404860] Law J. (2008). On sociology and STS. The Sociological Review, 56(4), 623–649. 10.1111/j.1467-954X.2008.00808.x

[bibr52-13684310251404860] Law J. (2015). What's wrong with a one-world world? Distinktion: Journal of Social Theory, 16(1), 126–139. 10.1080/1600910X.2015.1020066

[bibr53-13684310251404860] Law J. Mol A. (2002). Local entanglements or utopian moves: An inquiry into train accidents. In Parker M. (Ed.), Utopia and organization (pp. 82–105). Blackwell.

[bibr54-13684310251404860] Law J. Urry J. (2004). Enacting the social. Economy and Society, 33(3), 390–410. 10.1080/0308514042000225716

[bibr55-13684310251404860] Leary T. (1970). The politics of ecstasy. Paladin.

[bibr56-13684310251404860] Legacy C. Clements R. Woodcock I. Whitten J. (2024). Proposing an ethics of care: Tracing Victoria's transport planning history. Geographical Research, 62(3), 389–401. 10.1111/1745-5871.12663

[bibr57-13684310251404860] Lohrey A. Boreham B. (2020). The nonlocal universe. Communicative & Integrative Biology, 13(1), 147–159. 10.1080/19420889.2020.1822583 PMC7588183 33149801

[bibr58-13684310251404860] Lynch M. (2013). Ontography: Investigating the production of things, deflating ontology. Social Studies of Science, 43(3), 444–462. 10.1177/0306312713475925

[bibr59-13684310251404860] Lynch M. (2019). Ontography as the study of locally organized ontologies. ZMK Zeitschrift für Medien- und Kulturforschung, 10(1), 147–160. 10.28937/1000108239

[bibr60-13684310251404860] Manderscheid K. (2012). Automobilität als raumkonstituierendes Dispositiv der Modeme. In Füller H. Michel B. (Eds.), Die Ordnung der Räume (pp. 145–178). Westphälisches Dampfboot.

[bibr61-13684310251404860] Manderscheid K. (2014). The movement problem, the car and future mobility regimes: Automobility as dispositif and mode of regulation. Mobilities, 9(4), 604–626. 10.1080/17450101.2014.961257

[bibr62-13684310251404860] Martens K. (2016). Transport justice: Designing fair transportation systems. Routledge.

[bibr63-13684310251404860] Massumi B. (2015). Ontopower: War, powers, and the state of perception. Duke University Press.

[bibr64-13684310251404860] Maynard D. W. Heritage J. (Eds.). (2022). The ethnomethodology program: Legacies and prospects. Oxford Academic. 10.1093/oso/9780190854409.001.0001

[bibr65-13684310251404860] McGowan J. (2022). Hannah Arendt’s performative politics. In Gaonkar D. Topper K. (Eds.), The Oxford handbook of rhetoric and political theory. Oxford Academic. 10.1093/oxfordhb/9780190220945.013.29

[bibr66-13684310251404860] Merriman P. (2009). Automobility and the geographies of the car. Geography Compass, 3(2), 586–599. 10.1111/j.1749-8198.2009.00219.x

[bibr67-13684310251404860] Miner P. Smith B. M. Jani A. McNeill G. Gathorne-Hardy A. (2024). Car harm: A global review of automobility's harm to people and the environment. Journal of Transport Geography, 115, 103817. 10.1016/j.jtrangeo.2024.103817

[bibr68-13684310251404860] Mol A. (1999). Ontological politics: A word and some questions. The Sociological Review, 47, 74–89. 10.1111/j.1467-954X.1999.tb03483.x

[bibr69-13684310251404860] Mol A. Smith B. H. Weintraub E. R. (2002). The body multiple: Ontology in medical practice. Duke University Press. 10.1515/978082238415

[bibr70-13684310251404860] Newman D. (2013). Cars and consumption. Capital & Class, 37(3), 457–476. 10.1177/0309816813505164

[bibr71-13684310251404860] Norton P. D. (2008). Fighting traffic: The dawn of the motor age in the American city. MIT Press.

[bibr72-13684310251404860] Oksala J. (2010a). Foucault's politicization of ontology [article]. Continental Philosophy Review, 43(4), 445–466. 10.1007/s11007-010-9153-6

[bibr73-13684310251404860] Oksala J. (2010b). Foucault’s politicization of ontology. Continental Philosophy Review, 43(4), 445–466. 10.1007/s11007-010-9153-6

[bibr74-13684310251404860] Pollner M. (1974). Mundane reasoning. Philosophy of the Social Sciences, 4(1), 35–54. 10.1177/004839317400400103

[bibr75-13684310251404860] Pollner M. (1987). Mundane reason: Reality in everyday and sociological discourse. Cambridge University Press.

[bibr76-13684310251404860] Pollner M. (1991). Left of ethnomethodology: The rise and decline of radical reflexivity. American Sociological Review, 56(3), 370–380. 10.2307/2096110

[bibr77-13684310251404860] Prozorov S. (2017). Carl Schmitt. In Adam K. Carlo S. (Eds.), Agamben's philosophical lineage (pp. 87–98). EUP. https://library3.webster.edu/login?url=https://search.ebscohost.com/login.aspx?direct=true&db=nlebk&AN=1923829&site=eds-live&scope=site

[bibr78-13684310251404860] Randell R. Božič I. R. Braun R. Kotašková E. Nedbálková K. Němeček K. Paul T. Szaló C. (Forthcoming). Walking in the spatial order of automobility. In Snellgrove M. (Ed.), Leisured walking: Mobilities, encounters and critical engagements. Routledge.

[bibr79-13684310251404860] Randell R. Braun R. (2022). Automobility studies. In Glăveanu V. P. (Ed.), The Palgrave encyclopedia of the possible (pp. 151–157). Springer International Publishing. 10.1007/978-3-030-90913-0_11

[bibr80-13684310251404860] Raymond C. M. Rautio P. Fagerholm N. Aaltonen V. A. Andersson E. Celermajer D. Christie M. Hällfors M. Saari M. H. Mishra H. S. Lechner A. M. Pineda-Pinto M. Schlosberg D. (2025). Applying multispecies justice in nature-based solutions and urban sustainability planning: Tensions and prospects. npj Urban Sustainability, 5(1), 2. 10.1038/s42949-025-00191-2

[bibr81-13684310251404860] Rorty R. (1982). Philosophy as a kind of writing: An essay on Derrida. In Consequences of Pragmatism: Essays 1972-1980. University of Minnesota Press.

[bibr82-13684310251404860] Schmitt C. (2003). The nomos of the earth in the international law of the Jus Publicum Europaeum. Telos Press. (1950).

[bibr83-13684310251404860] Schmitt C. (2005). Political theology: Four chapters on the concept of sovereignty. University of Chicago Press.

[bibr84-13684310251404860] Seo S. A. (2019). Policing the open road: How cars transformed American freedom. Harvard University Press.

[bibr85-13684310251404860] Shapin S. (1984). Pump and circumstance: Robert Boyle’s literary technology. Social Studies of Science, 14(4), 481–520. 10.1177/030631284014004001

[bibr86-13684310251404860] Sheller M. (2018). Mobility justice: The politics of movement in an age of extremes. Verso.

[bibr87-13684310251404860] Sheller M. Urry J. (2006). The new mobilities paradigm. Environment and Planning A: Economy and Space, 38(2), 207–226. 10.1068/a37268

[bibr88-13684310251404860] Sheller M. Urry J. (2016). Mobilizing the new mobilities paradigm. Applied Mobilities, 1(1), 10–25. 10.1080/23800127.2016.1151216

[bibr89-13684310251404860] Smith D. E. (1999). Writing the social: Critique, theory, and investigations. Toronto University Press.

[bibr90-13684310251404860] Smith D. E. (2001). Texts and the ontology of organizations and institutions. Studies in Cultures, Organizations and Societies, 7(2), 159–198. 10.1080/10245280108523557

[bibr91-13684310251404860] Smith D. E. (2005). Institutional ethnography: A sociology for people. AltaMira.

[bibr92-13684310251404860] Toulmin S. E. (2013). Cosmopolis: The hidden agenda of modernity. Univ. of Chicago Press.

[bibr93-13684310251404860] Urry J. (2004). The ‘system’ of automobility. Theory, Culture & Society, 21(4-5), 25–39. 10.1177/0263276404046059

[bibr94-13684310251404860] Virilio P. (2007). The original accident. Polity Press.

[bibr95-13684310251404860] Viveiros de Castro E. (2011). Zeno and the art of anthropology. Common Knowledge, 17(1), 128–145. 10.1215/0961754X-2010-045

[bibr96-13684310251404860] Voelkner N. Zanotti L. (2022). Ethics in a quantum world. Global Studies Quarterly, 2(3), 1–5. 10.1093/isagsq/ksac044

[bibr97-13684310251404860] Wallace D. (2012). The emergent multiverse: Quantum theory according to the Everett interpretation. Oxford University Press.

[bibr98-13684310251404860] West C. Zimmerman D. H. (1987). Doing gender. Gender and Society, 1(2), 125–151. 10.1177/0891243287001002

[bibr99-13684310251404860] Wiley N. Garfinkel H. (2019). Interview with Harold Garfinkel. Human Studies, 42, 165–181. 10.1007/s10746-019-09517-3

[bibr100-13684310251404860] Wittgenstein L. (1953). Philosophical investigations. Macmillan.

[bibr101-13684310251404860] Woolgar S. (2022). The value of strident agnosticism: Dorothy Pawluch and the endurance of ontological gerrymandering. The American Sociologist, 53(1), 176–187. 10.1007/s12108-021-09524-x

[bibr102-13684310251404860] Woolgar S. Lezaun J. (2013). The wrong bin bag: A turn to ontology in science and technology studies? Social Studies of Science, 43(3), 321–340. 10.1177/0306312713488820

[bibr103-13684310251404860] Woolgar S. Pawluch D. (1985). Ontological gerrymandering: The anatomy of social problems explanations. Social Problems: Constructionist Readings, 32(3), 214–227. 10.2307/800680

[bibr104-13684310251404860] Zhang Y. Liu T. Bai Q. Shao W. Wang Q. (2018). New systems-based method to conduct analysis of road traffic accidents. Transportation Research Part F: Traffic Psychology and Behaviour, 54, 96–109. 10.1016/j.trf.2018.01.019

